# N-Acetylcysteine Attacks Phenoloxidase to Inhibit Insect Melanization and Boost Fungal Virulence

**DOI:** 10.3390/jof12090670

**Published:** 2026-09-07

**Authors:** Yingyu Tang, Yuyang Lian, Shirong Li, Erjun Ling, Dongzhi Wei, Wuren Huang

**Affiliations:** 1 State Key Laboratory of Bioreactor Engineering, New World Institute of Biotechnology, East China University of Science and Technology, Shanghai 200237, China; tangyingyu@cemps.ac.cn; 2 University of Chinese Academy of Sciences, Beijing 101408, China; 3 CAS Center for Excellence in Molecular Plant Sciences, Institute of Plant Physiology and Ecology, Chinese Academy of Sciences, Shanghai 200036, China; 4 Shaanxi Key Laboratory of Research and Utilization of Resource Plants on the Loess Plateau, College of Life Sciences, Yan’an University, Yan’an 716000, China

**Keywords:** N-acetylcysteine, *Drosophila*, *Beauveria bassiana*, fungal infection, prophenoloxidase, virulence

## Abstract

The prophenoloxidase (PPO) melanization cascade is a cornerstone of insect humoral immunity against fungal pathogens, yet its direct fungicidal mechanism remains undefined, and strategies to safely manipulate this pathway for biological control are lacking. Here, we demonstrate that infection of *Drosophila* by the entomopathogenic fungus *Beauveria bassiana* elicits a surge in phenoloxidase (PO) activity directly triggered by the fungal cell wall component β-1,3-Glucan. This activated PO generates reactive oxygen species (ROS) that are essential for restricting fungal proliferation, thereby establishing a mechanistic link between cascade activation and fungicidal outcome. Critically, we uncover that N-acetylcysteine (NAC), a safe, low-cost pharmaceutical in global clinical use, acts as a potent direct inhibitor of PO enzymatic activity, neutralizing the melanization-driven ROS burst. NAC-mediated inhibition significantly accelerates *B. bassiana* virulence and host mortality *in vivo*. Our work defines a new molecular function for NAC as a melanization inhibitor and reveals a safe, translatable strategy to suppress host immune defenses, offering a promising approach to enhance the efficacy of fungal biopesticides for sustainable pest control worldwide.

## 1. Introduction

The prophenoloxidase (PPO) cascade, a cornerstone of invertebrate innate immunity, drives the rapid melanization of pathogens and wounds [1,2,3]. Historically viewed as a broad-spectrum humoral defense, intriguingly, recent research reveals that PPO activation is a finely regulated process with distinct, pathogen-specific dynamics. This is particularly evident in antifungal defense, where melanization constitutes a critical frontline barrier [4,5]. Across diverse insect orders, including Lepidoptera, Diptera, and Coleoptera, PPO activation is a conserved and essential response to a wide range of entomopathogenic fungi such as *Beauveria bassiana* and/or *Metarhizium anisopliae* [4,5,6,7,8,9,10]. The central role of melanization in this defense is underscored by the observation that many successful pathogens have evolved specific counterstrategies, including the secretion of proteases that directly cleave and inactivate host PPO, to overcome this immune barrier [6,11].

The *Drosophila* Toll pathway and PPO-mediated melanization represent the crucial host immune responses against pathogenic fungi [1,3,5]. Biochemically, the proteolytic activation of PPO is integrated with the Toll pathway via shared upstream serine proteases, forming a coordinated defense network [12]. Notably, this integration includes a feedback loop wherein melanization byproducts modulate Toll signaling, revealing sophisticated immune cross-talk [5,12]. Genetic studies definitively establish that hemolymph PPOs are essential for survival against entomopathogenic fungi like *Beauveria bassiana*. While both PPO1 and PPO2 contribute, PPO2 plays a more dominant antifungal role, whereas PPO1 is crucial for antibacterial defense, highlighting functional specialization within the cascade [4]. Similarly, in the mosquito *Armigeres subalbatus*, two distinct PPO isoforms are differentially expressed in response to blood-feeding and filarial worm infection, suggesting that PPO functional specialization in response to different immune challenges is a broader phenomenon across insects [13,14]. The resistance function is highlighted by the uncontrolled fungal dissemination in Toll-pathway mutants [5]. Furthermore, the strategic importance of this defense depends strongly on the infection route. Melanization proves most critical during natural topical infection, forming an early barrier at the cuticle, a finding that underscores its evolutionary role in resisting the most common route of fungal attack [5,6,15]. In response, successful fungal pathogens have evolved elegant countermeasures. Some adopt a stealthy, cell-wall-free protoplast form to evade immune recognition entirely [5]. Others, like *B. bassiana*, secrete proteases such as BPS8 that cleave and inactivate host PPO, directly inhibiting this defense [6]. However, it remains unclear how PPO-mediated melanization kills invading fungi in *Drosophila* and whether this process can be utilized for pest biocontrol.

Here, we demonstrate that *B. bassiana* infection triggers a surge in PO activity without depleting PPO protein expression, a process directly elicited by fungal β-1,3-Glucan. This activated melanization drives the production of reactive oxygen species (ROS) that are toxic [16,17,18]. Strikingly, we identify N-acetylcysteine (NAC), a safe low-cost antioxidant [19], as a potent direct inhibitor of PO activity *in vivo*. By suppressing this key immune arm, NAC dramatically accelerates fungal virulence. Our work not only elucidates the effector mechanism of antifungal melanization but also reveals a transformative strategy: pairing NAC with entomopathogenic fungi to create fast-acting mycoinsecticides. The aim of this study is twofold: first, to elucidate the direct effector mechanism by which PPO-mediated melanization exerts its fungicidal activity during *B. bassiana* infection in *Drosophila*, and second, to test the hypothesis that safe, small-molecule inhibitors can strategically suppress this immune arm to dramatically accelerate fungal virulence for biopesticide development.

## 2. Material and Methods

### 2.1. Drosophila Stocks and Rearing

The fly lines utilized in this study were as follows: *w^1118^* (BDSC_3605) and *PPO1*^Δ^, *PPO2*^Δ^ [4]. Fly stocks were reared and maintained on a standard food (1 L food containing 5% cornmeal, 5% yeast, 0.5% agar, and 0.285% Tegosept). Prior to infection, adult flies were transferred to their respective media, supplemented with antibiotics, and maintained on these diets for two days (see following section). These experimental diets were Tegosept-free and stored at 4 °C for 1 week to prevent microbial contamination. Each experiment was replicated at least three times, and a representative set of data was presented.

### 2.2. Fungi and Infection

Prior to fungal infection, female flies were transferred to their respective food containing antibiotics, formulated based on a published paper with some modifications [20], and maintained for two days to generate gnotobiotic adults. *Beauveria bassiana ARSEF 2860* [21] was routinely cultured at 25 °C for 10 days. Conidia (spores) of *B. bassiana* were then collected. Following a previously established protocol [6], these conidia were resuspended in 0.05% (*v*/*v*) Tween 20 at concentrations of 1 × 10^7^ conidia/mL for infection of adult *Drosophila*. Infected insects were monitored every 12 h, and mortality was recorded as part of the bioassay. For *Drosophila*, adults or specific tissues or plasma collected on day 4 were used for Western blot unless otherwise noted.

### 2.3. Fungal Load

Fungal genomic DNA was extracted from infected flies and dissected tissue samples of infected insects following a previously established protocol [22]. Purified DNA was quantified, aliquoted, and stored at −80 °C for subsequent quantitative real-time PCR assays. For the detection of *B. bassiana*, primers for fungal load quantification were designed targeting a specific beta-tubulin gene sequence of *B. bassiana* (Bbtub, BBA_07018), which was screened and verified via nucleotide BLAST (+ 2.14.0) analysis. Fungal abundance was normalized to the *Drosophila* *ribosomal* *protein L32 gene* (*DmRpl32*, *CG7939*), with results presented as the BbTub/DmRpl32 ratio to represent fungal load. All samples were analyzed in triplicate, and the normalized datasets were applied to quantify relative fungal DNA levels.

### 2.4. Western Blot Assay

Following infection with *Beauveria bassiana*, adult insects or isolated tissues were harvested at predetermined time points. Samples were dissected, rinsed three times with 0.85% NaCl solution to eliminate hemolymph proteins, and lysed in 100 μL RIPA Lysis Buffer (Cat. No. P0013B, Beyotime Biotechnology, Shanghai, China). All samples were subsequently sonicated on ice and centrifuged at 10,000× *g* for 10 min at 4 °C. The collected supernatant was mixed with 5× SDS loading buffer and incubated at 100 °C for 5 min. The prepared samples were then subjected to Western blot analysis. The primary antibodies used in this study included β-Actin Rabbit Monoclonal Antibody (High Dilution) (Cat. No. AC026, ABclonal, Wuhan, China), as well as *Drosophila* PPO1 and PPO2 antibodies [23,24]. The secondary antibody adopted was HRP-conjugated goat anti-rabbit IgG (H + L) (Cat. No. AS029, ABclonal, Wuhan, China). Protein bands were visualized using ECL Plus reagent (Cat. No. E423-01/02, Vazyme, Nanjing, China). Band intensity was quantified and normalized to the β-Actin internal control. At least three biological replicates were included for each sample to enable statistical analysis.

### 2.5. Phenoloxidase Activity

The plasma, freshly prepared as described above, was used to assay PO activity. Briefly, approximately 2 μL of plasma was mixed with 200 μL of 10 mM dopamine (Sigma, Shanghai, China, H8502) dissolved in PBS buffer and incubated at room temperature for 10 min. The absorbance was measured at 470 nm using an EXPERT 96 microplate reader (Biochrom, Shanghai, China). One unit of PO activity was defined as △A470/min/plasma volume = 0.001.

### 2.6. Injection of β-1,3-Glucan, β-D-Glucan, β-Glucan and Chitin

β-Glucan (Aladdin, Shanghai, China, G304913), β-1,3-Glucan (Sigma, Shanghai, China, 89862), β-D-Glucan (Aladdin, Shanghai, China, G1210600), and Chitin (Coolaber, Beijing, China, V900332) were prepared in sterile 0.85% NaCl solution to a final concentration of 40 μg/μL. Approximately 69 nl of each prepared solution was injected into 3–7-day-old adult flies using the Nanoject II AUTOMATIC NANOLITER INJECTOR (3-000-205A, Drummond Scientific, Broomall, PA, USA). All adults were anesthetized on ice prior to injection. Following microinjection, the flies were kept on ice for 30 min and then returned to regular food. Once all individuals fully recovered, samples were collected at 12 h post treatment for subsequent detection.

### 2.7. Reactive Oxygen Species (ROS) Analysis

Fat bodies were dissected without any buffer to avoid loss of ROS, and subsequently stained with dihydroethidium (DHE; Thermo Fisher Scientific, Shanghai, China) diluted 1:2000 in 0.85% NaCl. Staining was performed for 5–10 min in a clean humid chamber, after which samples were rinsed with 0.85% NaCl solution. Specimens were subsequently mounted on slides in 0.85% NaCl and immediately examined via fluorescence microscopy.

### 2.8. Fungal Cell Viability

**Figure 4:**
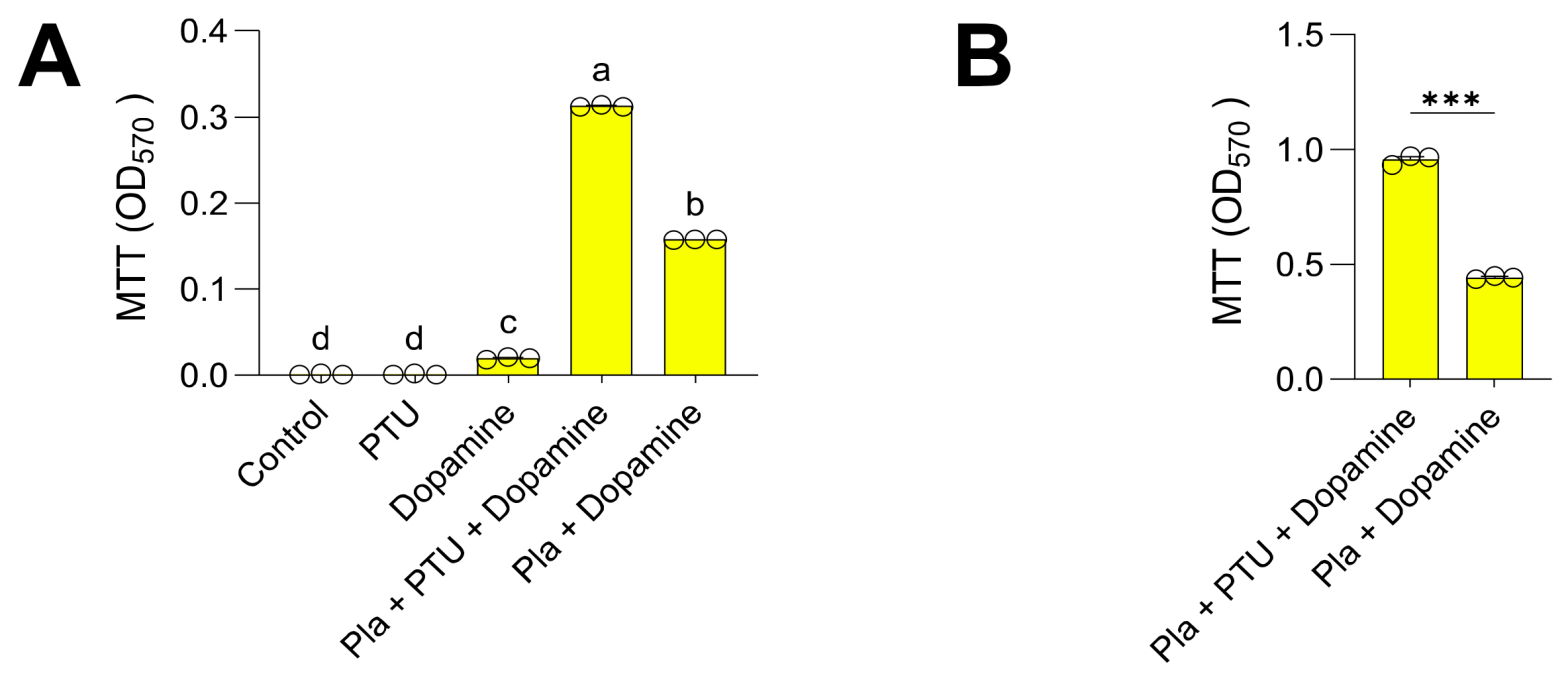
PO-induced melanization is toxic to *B. bassiana* spores and hyphae. (**A**) Toxicity to spore germination and growth. Spores were cultured with the indicated components, and metabolic activity (viability) was assessed by MTT assay. PO-activated plasma plus dopamine inhibits growth, an effect reversed by the PO inhibitor PTU. (**B**) Toxicity to hyphae. Hyphae, pre-grown from spores for 12 h, were treated similarly. Consistently, PO-mediated melanization inhibited hyphal metabolism, and this inhibition was alleviated by PTU. Pla, plasma. The dots (mean ± SEM) are representative of at least three independent experiments. Welch’s *t*-test (**B**); two-way ANOVA (**A**). ***, *p* < 0.001; a–d, significant difference, *p* < 0.05.

Approximately 1000 spores were suspended in 500 μL of 10% diluted Sabouraud Dextrose Broth (SDB) medium. The following materials were added to each 1.5 mL tube as required: 1 μL of saturated phenylthiourea (PTU) solution, 1 μL of 10 mM dopamine stock solution, and 10 μL of plasma containing activated PO (see Figure 4A). *B. bassiana* cultures were incubated at 28 °C for 12 h. To assess the impact of melanization on hyphal viability, spores were first cultured in 500 μL of SDB medium for 24 h, followed by the addition of PTU, dopamine, and plasma as described above for another 12 h culture. All tubes were then centrifuged at 10,000 rpm for 5 min at room temperature. Supernatants were discarded, and the pellets were washed once with PBS buffer and suspended in 100 μL of PBS buffer. Fungal cell viability was evaluated upon adding 5 μL of 3-(4,5-dimethylthiazol-2-yl)-2,5-diphenyltetrazolium bromide (MTT, 5 mg/mL prepared in PBS) to each tube. The tubes were incubated at 37 °C for 4 h. The supernatant was subsequently discarded after centrifugation, and the formed insoluble formazan precipitate was fully dissolved with 200 µL of dimethyl sulfoxide (DMSO). The optical absorbance of each sample was recorded at a wavelength of 570 nm.

### 2.9. Fly Adult Plasma Collection

To collect plasma, the thoraxes of approximately 60 flies were pierced using a tungsten needle. The treated flies were then transferred into a perforated 0.5 mL Eppendorf tube, which was nested inside a 1.5 mL Eppendorf tube. Samples were centrifuged twice at 3000× *g* for 4 min at 4 °C, with gentle agitation of the flies between the two centrifugation steps. The harvested hemolymph was subsequently centrifuged at 10,000× *g* for 15 min to eliminate insoluble components. Finally, the processed hemolymph was stored at −80 °C prior to the PO activity assay.

### 2.10. Influence of N-Acetylcysteine on PO Activity and Fungal Virulence Assessment

In a total volume of 200 μL of 10 mM dopamine pre-supplemented with NAC to achieve different final concentrations, 1 μL of plasma was collected from silkworm larvae or fly adults, followed by another 10 min incubation at room temperature for subsequent PO activity measurement.

Fly food was prepared by incorporating NAC to a final concentration of 14 mM prior to cooling. Following *B. bassiana* infection, both infected and uninfected control flies were transferred to the NAC-supplemented diet. On day 4 post infection, samples were harvested for the detection of ROS levels, PO activity, and PPO protein abundance in accordance with the above protocol.

### 2.11. Quantification and Statistical Analysis

Fluorescence intensity was measured from acquired images using ImageJ (1.8.0.345) software (National Institutes of Health, Madison, WI, USA). All images were acquired under identical experimental conditions within each experimental set. Statistical significance was assessed by Welch’s *t*-test or one-/two-way ANOVA as appropriate. Unless stated otherwise, the results are representative of at least three independent experiments.

## 3. Results and Discussion

### 3.1. B. bassiana Infection Enhances Phenoloxidase Activity Without Altering Prophenoloxidase Protein Levels

**Figure 1:**
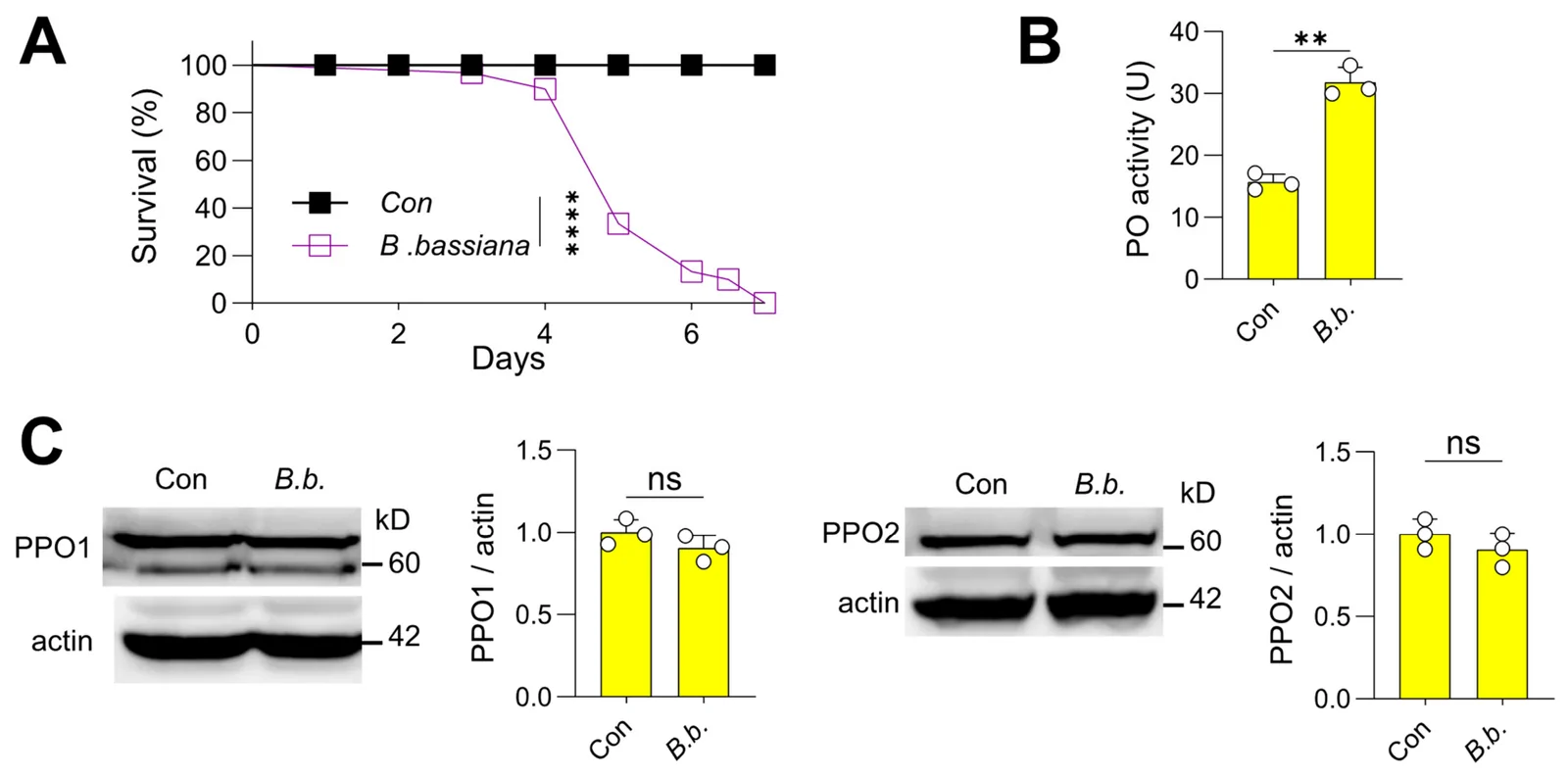
*B. bassiana* infection enhances PO activity without affecting PPO1 and PPO2 expression. (**A**) Survival of *Drosophila* adults after topical infection with *B. bassiana*. (**B**) PO activity is enhanced by fungal infection. Plasma was collected on day 4 post infection for the PO activity assay. (**C**) PPO1 and PPO2 protein levels are unaffected by infection. Whole-body lysates were analyzed by Western blot. Con, control. *B.b.*, *B. bassiana*. The dots (mean ± SEM) are representative of at least three independent experiments. Welch’s *t*-test (**B**,**C**). **, *p* < 0.01; ****, *p* < 0.0001; ns, not significant.

*B. bassiana* spores were topically applied to *Drosophila* adults as previously described [6]. By day 4, when the adults began to die (Figure 1A), plasma activated phenoloxidase (PO) activity was significantly upregulated (Figure 1B). Concurrently, the levels of *Drosophila* prophenoloxidase (PPO) proteins, PPO1 and PPO2, remained essentially unchanged (Figure 1C). Moreover, identical trends for PO activity and PPO levels were observed throughout the entire infection course. Ultimately, infected adults succumbed to *B. bassiana* infection. Collectively, these data demonstrate that *B. bassiana* infection enhances plasma PO activity without reducing PPO protein levels. This uncoupling of PO activity from PPO protein abundance reveals that infection triggers rapid, post-translational activation of a pre-existing latent pool, representing an economical and evolutionarily conserved strategy to mount an immediate, yet sustained, enzymatic immune response without the energetic cost of de novo synthesis.

### 3.2. β-1,3-Glucan and β-Glucan Are the Primary Fungal Elicitors of Enhanced PO Activity

**Figure 2:**
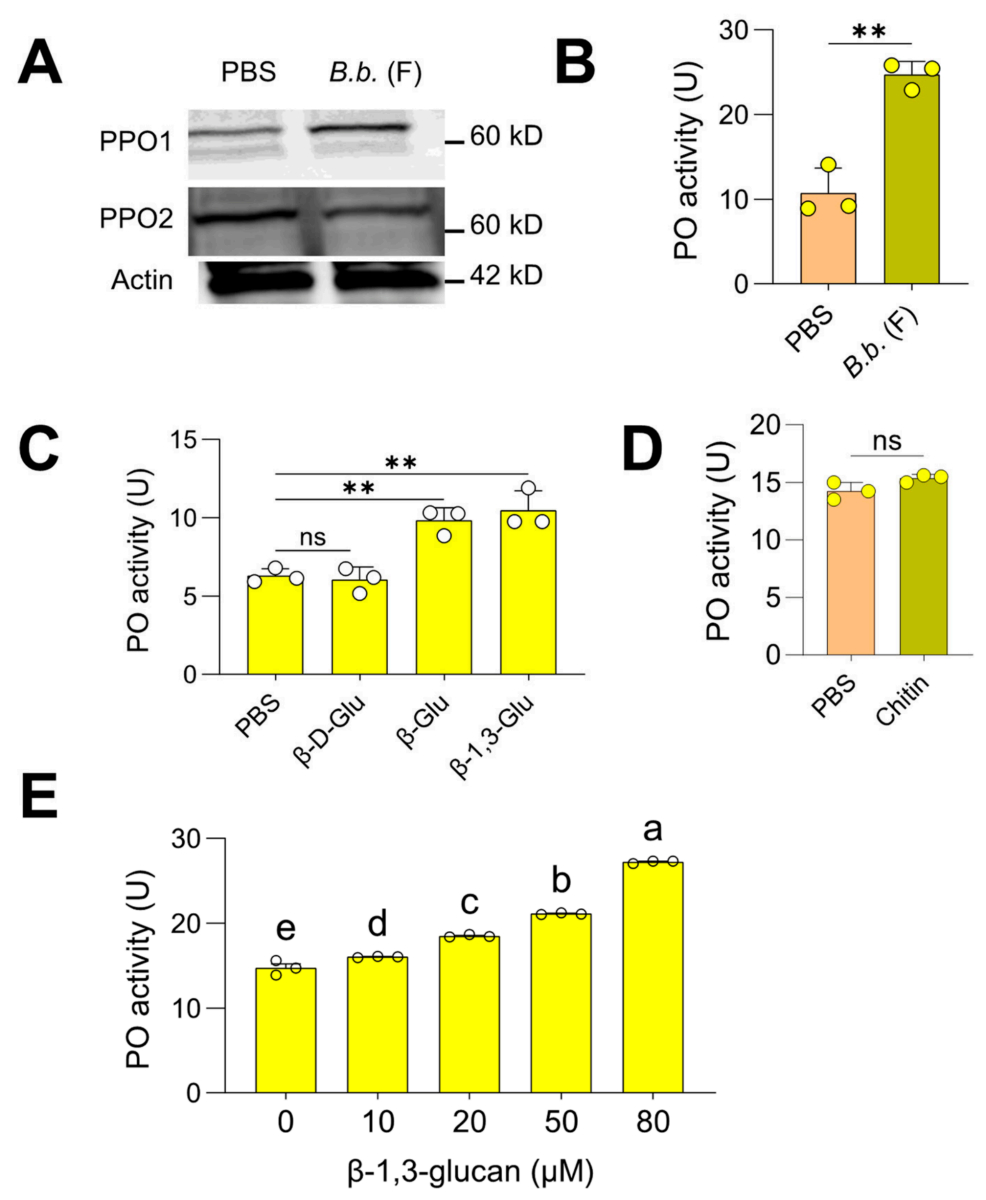
β-1,3-Glucan and β-Glucan are key fungal elicitors of PO activity. (**A**,**B**) Injection of formalin-killed *B. bassiana* spores enhances PO activity without altering PPO protein levels. Adults were injected with 4 × 10^9^ spores, and PPO1/PPO2 levels (**A**) and plasma PO activity (**B**) were assessed 16 h later. (**C**,**D**) Identification of the specific fungal component responsible for *Drosophila* PPO activation. The indicated carbohydrates were injected. (**C**) PO activity was measured 16 h after injecting various Glucans; β-1,3-Glucan and β-Glucan, but not β-D-Glucan, enhanced activity. (**D**) Injection of chitin did not alter PO activity. (**E**) β-1,3-Glucan activates PO in *Drosophila* adult plasma in a dose-dependent manner in vitro. PO activity was measured after incubating diluted plasma with the indicated concentrations of β-1,3-Glucan for 5 min. *B.b.* (F), formalin-killed *B. bassiana* spores. Glu, Glucan. The dots (mean ± SEM) are representative of at least three independent experiments. Welch’s *t*-test (**B**,**D**); two-way ANOVA (**C**,**E**). **, *p* < 0.01; ns, not significant; a–e, significant difference, *p* < 0.05.

We next injected formalin-killed *B. bassiana* spores into adult flies. Notably, PPO1 and PPO2 levels remained unchanged (Figure 2A), whereas PO activity was significantly upregulated (Figure 2B). This finding indicates that formalin-inactivated *B. bassiana* spores contain bioactive factors capable of activating plasma PPO in *Drosophila*. The fungal membrane surface is rich in carbohydrates such as β-1,3-Glucan and chitin [25,26]. Therefore, we injected different Glucans into adult flies. The results revealed that β-Glucan and β-1,3-Glucan, but not β-D-Glucan, significantly enhanced PO activity (Figure 2C). Additionally, although the fungal surface also contains abundant chitin [25,26], chitin injection did not alter PO activity (Figure 2D). *In vitro*, β-1,3-Glucan activated PO activity in naïve adult fly plasma in a dose-dependent manner (Figure 2E). Collectively, these findings indicate that fungal membrane β-1,3-Glucan is a direct, dose-dependent enhancer of *Drosophila* PO activity. This functional identification of β-1,3-Glucan as the key pathogen-associated molecular pattern (PAMP) driving melanization provides a critical mechanistic link: the host innate immune system directly decodes a conserved fungal surface signature to deploy a specific and scalable enzymatic defense, a principle that echoes pattern recognition in vertebrate immunity.

### 3.3. PO Activity Directly Drives Reactive Oxygen Species Production During Fungal Infection

**Figure 3:**
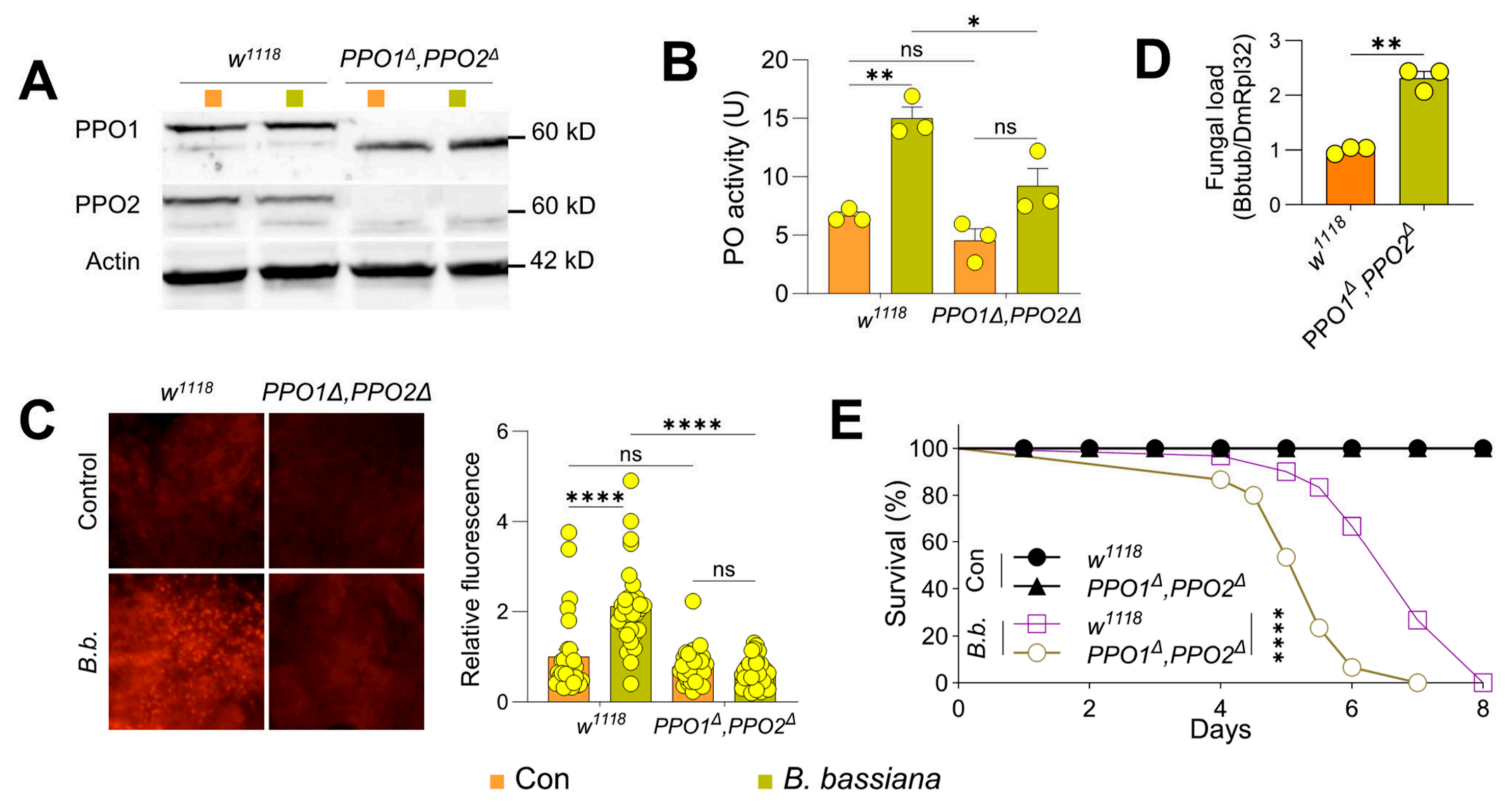
PPO is required for ROS induction after *B. bassiana* infection. (**A**,**B**) PPO expression and PO activity in the *PPO1^Δ^, PPO2^Δ^* mutant. Wild-type (*w^1118^*) and mutant adults were infected, and PPO1/PPO2 levels (**A**) and PO activity (**B**) were analyzed on day 4 post infection. (**C**) PPO is required for infection-induced ROS production. Fat bodies from infected flies of the indicated genotypes were dissected on day 4 post infection for ROS staining. Representative images (**left**) and quantification (**right**) are shown. (**D**) Fungal burden is higher in the *PPO1^Δ^, PPO2^Δ^* mutant. Fungal load was quantified in cadavers under identical conditions. (**E**) The *PPO1^Δ^, PPO2^Δ^* mutant exhibits accelerated mortality following topical *B. bassiana* infection. Con, control. *B.b.*, *B. bassiana*. The dots(mean ± SEM) are representative of at least three independent experiments. Welch’s *t*-test (**D**); two-way ANOVA (**B**,**C**). *, *p* < 0.05; **, *p* < 0.01; ****, *p* < 0.0001; ns, not significant.

The *PPO1*^Δ^, *PPO2*^Δ^ mutant lacks expression of PPO1 and PPO2 proteins due to deletion of both genes [4]. After fungal infection, neither PPO1 nor PPO2 was detected in naive or infected *PPO1*^Δ^, *PPO2*^Δ^ mutants (Figure 3A). Correspondingly, PO activity in the mutant was comparable to that in naive wild-type (*w^1118^*) adults (Figure 3B). We then dissected fat bodies for reactive oxygen species (ROS) staining (Figure 3C). Quantification revealed that ROS levels in infected *w^1118^* flies were significantly higher than those in infected *PPO1*^Δ^, *PPO2*^Δ^ mutants and in both naive lines (Figure 3C). Furthermore, fungal load assays in *B. bassiana*-killed adults under the same conditions showed that the fungal burden in *PPO1*^Δ^, *PPO2*^Δ^ mutant flies exceeded that in *w^1118^* (Figure 3D). Consistently, *PPO1*^Δ^, *PPO2*^Δ^ mutants were indeed killed at a faster rate (Figure 3E), which is consistent with previous studies [4,5]. Together, these results indicate that ROS generated by elevated PO activity are important for antifungal defense. By genetically uncoupling the PPO-ROS axis, we provide the first causal in vivo evidence that melanization kills fungi through an ROS-mediated effector mechanism, transforming our understanding from a correlative defense response to a defined cytotoxic pathway that can be precisely targeted for immune manipulation.

### 3.4. PO-Induced Melanization Significantly Inhibits B. bassiana Growth

To further confirm the role of ROS produced by PO-induced melanization in restricting fungal growth, we cultured *B. bassiana* spores and hyphae in diluted SDB medium containing PO-activated plasma and dopamine. Larval plasma provides proteins and glucose that *B. bassiana* can utilize for growth. Consequently, fungi grown in medium containing plasma and dopamine proliferated significantly faster than those in groups with PTU or dopamine alone (Figure 4A). However, when phenylthiourea (PTU), an inhibitor of PO activity [1], was added to the plasma–dopamine mixture, *B. bassiana* grew faster than in the absence of PTU, indicating that PO-induced melanization suppresses fungal growth. Similarly, PTU-mediated inhibition of PO activity also promoted hyphal growth (Figure 4B). Overall, *B. bassiana* infection triggers host PPO activation, which in turn enhances PO activity, leading to elevated ROS levels that inhibit fungal growth. These *ex vivo* reconstitution experiments directly prove that the melanization reaction itself, not merely the physical melanin deposit, is toxic to fungi via a diffusible ROS burst, establishing a complete mechanistic chain from PAMP recognition to microbicidal effector, and pinpointing PO as the central enzymatic node for therapeutic intervention to enhance biocontrol.

### 3.5. N-Acetylcysteine Enhances Fungal Virulence by Directly Inhibiting PO Activity

**Figure 5:**
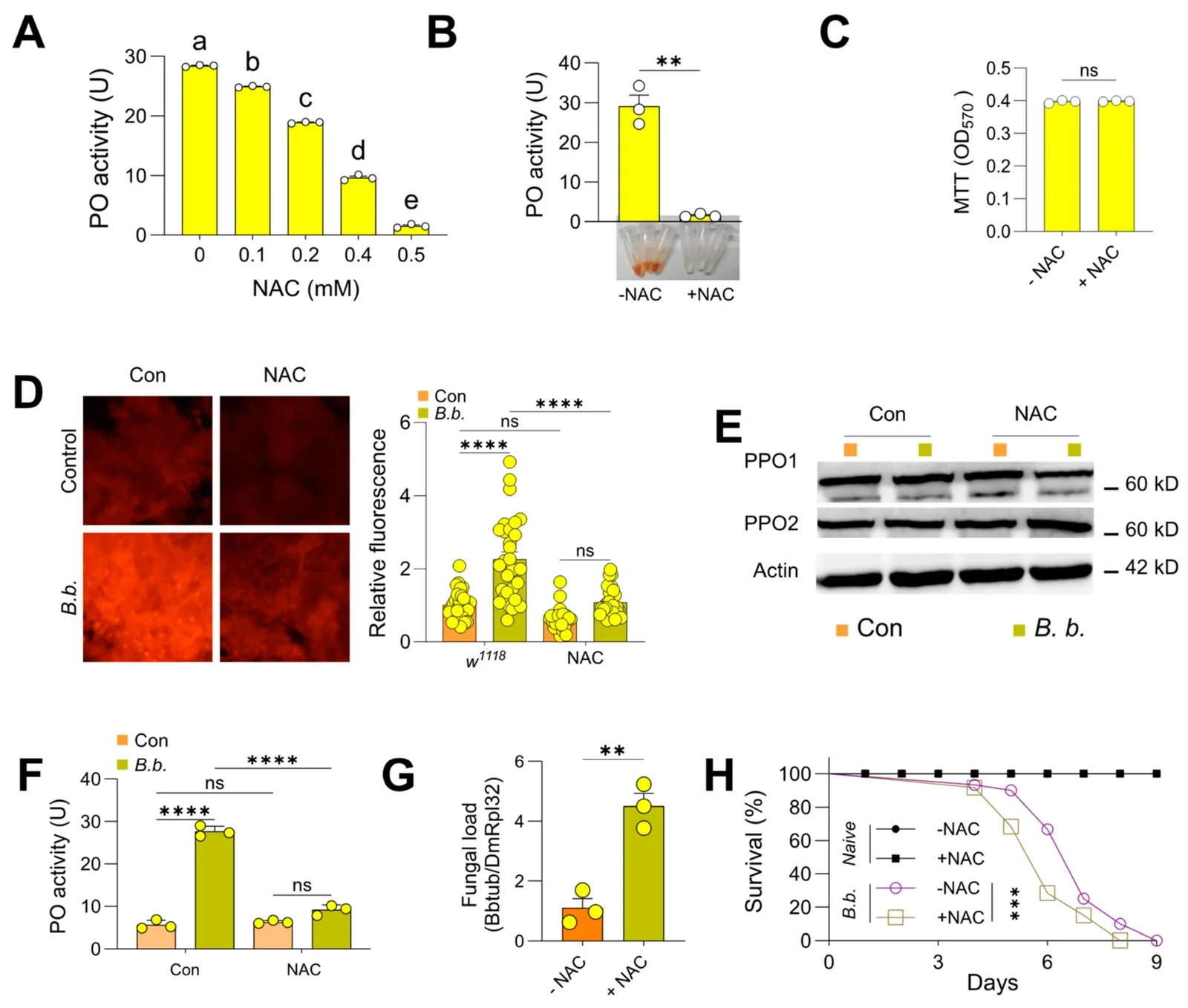
NAC inhibits host PO activity and ROS production to enhance fungal virulence. (**A**) NAC inhibits PO activity dose-dependently in vitro. PO-activated plasma was incubated with the indicated NAC concentrations prior to substrate addition. (**B**) NAC inhibits PO activity in plasma from infected flies. PO activity (**top**) and the visual outcome of dopamine oxidation (**bottom**) are shown. (**C**) NAC does not inhibit *B. bassiana* growth in vitro. Spore cultures were grown with or without 0.5 mM NAC. (**D**) Dietary NAC suppresses infection-induced ROS. Infected adults (*w^1118^*) were fed NAC-supplemented food. Fat body ROS was assessed and quantified on day 4 post infection. (**E**,**F**) Dietary NAC did not affect PPO1/PPO2 protein levels (**E**) but significantly inhibited PO activity (**F**). (**G**) Dietary NAC increases fungal burden. Fungal load was measured in fungus-killed adults fed with (+) or without (-) NAC supplementation. (**H**) NAC enhances *B. bassiana* virulence. Survival of infected adults was monitored on food with or without NAC supplementation. Con, control. *B.b.*, *B. bassiana*. The dots (mean ± SEM) are representative of at least three independent experiments. Welch’s *t*-test (**B**,**C**,**G**); two-way ANOVA (**A**,**D**,**F**). **, *p* < 0.01; ***, *p* < 0.001; ****, *p* < 0.0001; ns, not significant; a–e, significant difference, *p* < 0.05.

N-acetylcysteine (NAC) is widely used as a reactive oxygen species (ROS) scavenger in both invertebrate and vertebrate animals [19]. Intriguingly, we found that NAC directly inhibited PO activity in activated silkworm larval plasma in a dose-dependent manner (Figure 5A); at higher concentrations, PO activity was almost completely abolished. Correspondingly, NAC also suppressed PO activity in fungus-activated adult fly plasma (Figure 5B). Notably, without NAC, the substrate turned brown due to PO-mediated dopamine oxidation, whereas with NAC, no color change occurred. Unlike toxic PTU, NAC at 0.5 mM did not affect *B. bassiana* growth in culture (Figure 5C). Subsequently, fly food supplemented with approximately 0.5 mM NAC was fed to adults after fungal infection. ROS levels in fat bodies were significantly suppressed in the NAC-fed group (Figure 5D). Importantly, NAC ingestion exerted no discernible effect on the expression levels of PPO1 and PPO2 (Figure 5E), yet significantly suppressed in vivo PO activity (Figure 5F). Consistently, fungal load was significantly higher when the fly diet contained NAC (Figure 5G), and *B. bassiana* killed the host at a significantly faster rate than with control food (Figure 5H). Collectively, these results demonstrate that NAC application enhances *B. bassiana* virulence through direct inhibition of PO activity. PTU is toxic to animals when ingested [27,28,29]. NAC is extensively applied to the treatment of a broad spectrum of diseases, acting either alone or in combination with other pharmacological agents [19]. Notably, NAC may be exploited to enhance *B. bassiana* virulence for biological pest control due to its safety to animals.

In *Drosophila*, the Toll pathway-mediated production of antimicrobial peptides and prophenoloxidase (PPO)-dependent melanization are two fundamental innate immune responses against pathogenic fungal infections [1,2,3]. Notably, while the role of the Toll pathway is well-characterized, the precise fungicidal mechanism of PPO-mediated melanization remains largely elusive. Furthermore, a critical unanswered question is whether this potent immune mechanism can be effectively harnessed for the biological control of insect pests [1,30].

Biopesticides are increasingly vital for sustainable agriculture, yet each major class has inherent limitations that constrain their field efficacy. Entomopathogenic fungi, such as *B. bassiana* and *Metarhizium anisopliae*, are particularly promising because they infect via the cuticle, making them effective against most pests [31,32,33]. However, their main drawback is a relatively slow killing time, during which melanization, a key component of the insect humoral immune response, can reduce fungal establishment. Our finding that NAC directly inhibits PO activity, the central enzyme of melanization, suggests that it represents a novel strategy to overcome this barrier. NAC, a safe low-cost drug already approved for human use [19], can be applied alongside fungal spores to inhibit the host immune system, accelerating fungal proliferation and mortality. Unlike broad-spectrum immunosuppressants such as PTU (which is toxic to animals [27,28,29]), NAC is non-toxic and environmentally benign [19]. This principle may extend to other compounds that target PO, for example, small-molecule inhibitors of serine proteases upstream of the PO cascade, or RNAi-based suppressors of PPO expression [1,11]. Such immune-disrupting agents could be formulated with fungal spores to produce a new generation of fast-acting, high-efficacy mycoinsecticides. Future work should evaluate field performance, environmental fate, and non-target effects of NAC–fungus combinations. If successful, this strategy could transform fungal biopesticides from a niche product into a mainstream tool for integrated pest management.

## 4. Conclusions

In this study, we elucidate a critical effector mechanism of the insect melanization immune response, demonstrating that the direct generation of reactive oxygen species (ROS) by activated PO is essential for restricting *B. bassiana* proliferation, thereby providing a mechanistic link between PPO cascade activation and its fungicidal outcome. Concurrently, we uncover an unexpected function of NAC, a safe, low-cost pharmaceutical widely available across healthcare systems in regions from North America and Europe to Asia and Africa, as a direct inhibitor of PO activity. By efficiently suppressing the PO-driven ROS burst without toxic side effects, NAC effectively disables a key host defense, creating a permissive environment for fungal growth. This discovery repurposes NAC from a general antioxidant to a specific immune modulator, offering a proof of concept for a novel and safe strategy to enhance the virulence of entomopathogenic fungi. Unlike broad-spectrum and often toxic immunosuppressants, NAC’s mechanism is specific and its safety profile is well-established globally. We conclude that incorporating NAC, or potentially other specific PO inhibitors, into mycoinsecticide formulations represents a promising, translatable approach to overcome the critical limitation of slow killing speed in fungal biocontrol, a challenge recognized in both developed agricultural systems and developing nations reliant on cost-effective pest management. This strategy could significantly improve the field efficacy of fungal biopesticides, transforming them into a more powerful and sustainable tool for integrated pest management worldwide.

## Data Availability

All datasets generated and analyzed in this study are available from the corresponding authors upon reasonable request.
